# Successful rescue using tracheal intubation cannula for severe central airway stenosis after tracheotomy: A CARE compliant case report

**DOI:** 10.1097/MD.0000000000020117

**Published:** 2020-05-22

**Authors:** Hai-Feng Li, Bao-Peng Xing, Li-Sha Song, Wei Wang, Bao-Hua Liu

**Affiliations:** a Department of Critical Care Medicine, the First Hospital of Jilin University, Jilin, China; b Department of Hematopathology, University of Texas MD Anderson Cancer Center, Houston, TX; c Emergency Department, the First Hospital of Jilin University, Jilin, China.

**Keywords:** catheter, central airway stenosis, tracheal intubation, tracheotomy

## Abstract

**Introduction:**

Central airway stenosis is a life-threating requiring immediate medical intervention. There are several options for treating central airway stenosis, including rigid bronchoscopy, bronchoscopic high-power laser therapy, high-frequency electric needle knife, and balloon-expanding stents. However, interventional techniques may be unavailable in an emergent situation or at smaller local hospitals. In this case report, we publicly demonstrate for the first time that a tracheal intubation catheter may be applied as a temporary alternative to interventional bronchoscopic treatment.

**Patient concerns:**

A 72-year old male patient was admitted with a 1-year history of intermittent dyspnea, which was exacerbated for one day. One day prior to admission to our hospital, the patient presented with cyanosis due to an exacerbation of dyspnea.

A tracheotomy was performed and the patient had been carrying a tracheotomy cannula for 6 months.

**Diagnosis:**

The ventilator alarm indicated high airway resistance and the nurses were unable to insert the suction pipes into the airway. Immediate fiberoptic bronchoscopy showed diffuse edema and stenosis of the inferior tracheal airways.

**Interventions:**

Tracheal intubation was used to temporarily replace the tracheotomy cannula, which successfully expanded the narrowed airways.

**Outcomes:**

The blood oxygen saturation returned to normal, and dyspnea was quickly relieved.

**Conclusion:**

In emergent situations, tracheal intubation catheters may be used in patients with post-tracheotomy central airway stenosis, not only for surviving the most dangerous phase but for also prolonging the survival time for further treatments.

## 1 Core tip

Some long-term indwelling tracheostomy patients easily combined central airway stenosis, the serious airway obstruction can be life-threatening. At this point, the temporary application endotracheal intubation replace trachea cut catheter, using length of endotracheal intubation around and open obstruction, which make the remission, win precious time for the next treatment.

## 2 Introduction

Tracheostomy is a common procedure for long-term airway management. However, it is associated with a greater than 50% overall complication rate. Central airway stenosis is a potentially life-threatening complication associated with the presence of bacterial biofilms, increased bacterial counts, and the overexpression of specific inflammatory cytokines.^[1]^ It represents a therapeutic challenge and often requires interventional bronchoscopic treatment.^[2]^ However, interventional techniques may be unavailable in an emergent situation or at smaller local hospitals. In this case report, we demonstrate that a tracheal intubation catheter may be applied as a temporary alternative to interventional bronchoscopic treatment.

## 3 Case presentation

A 72-year old male patient was admitted with a 1-year history of intermittent dyspnea, which was exacerbated for 1 day. The patient was diagnosed with pulmonary heart disease at the local hospital 1 year prior to this incident. Since then, long-term oxygen therapy has been performed. Six months prior to this incident, the patient was treated in our department for dyspnea and unconsciousness. A tracheotomy was performed and the patient had been carrying a tracheotomy cannula for 6 months. One day prior to admission to our hospital, the patient presented with cyanosis due to an exacerbation of dyspnea. The patient's caretakers noticed increasing difficulty in sputum aspiration. In addition, the patient had a prior history of chronic obstructive pulmonary emphysema for more than 30 years, bilateral pulmonary bullae for more than 20 years, cerebral infarction 1 year earlier, and an old myocardial infarction without stent implantation and drug application for 2 years.

The patient was examined in the emergency department. Vital signs: temperature 36.5°C, heart rate 130 beats per minute, respiratory rate 30 times per minute, blood pressure 145/97 mmHg, and blood oxygen saturation 60%. The physical examination showed that the patient was obese and looked sick with signs of cyanosis, wet rales in the bilateral lungs, pitting edema of the lower extremities, and sluggish pupillary light reflex. Arterial blood gas results: PO_2_ 69.1 mmHg, Hct 60.2, Na^+^ 133.7 mmol/L, K^+^ 4.98 mmol/L, Ca^2+^ 1.008 mmol/L, Glu 7.1 mmol/L, Lac 1.4 mmol/L, tHb 166.3 g/L, HCO_3_^−^ 31.4 mmol/L, and PCT <0.10 μg/L.

**Figure 1:**
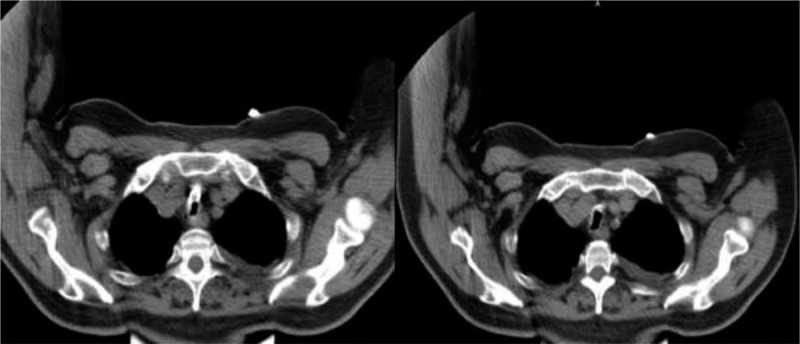
CT scan (mediastinal window) of this 72-year old male patient showed signs of tracheal stenosis, as indicated by the white arrow.

Chest computed tomography (CT) imaging revealed narrowing of the trachea, bilateral exudative lesions, and bilateral pulmonary bullae (Fig. 1). The patient was diagnosed of pneumonia. The complete medical diagnosis was an acute exacerbation of chronic obstructive pulmonary disease (COPD), double lung pneumonia, pulmonary bullae, and type II respiratory failure, post-tracheotomy.

**Figure 2:**
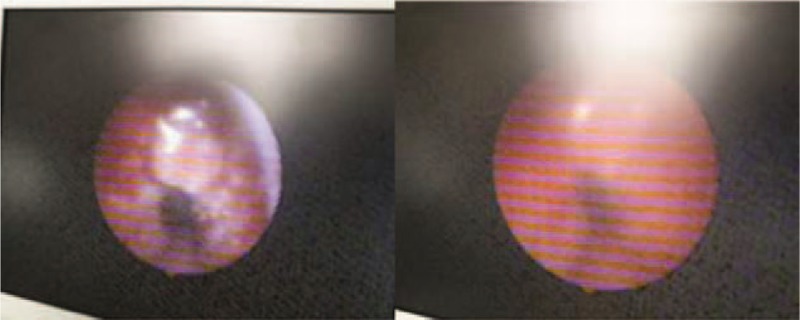
Bronchoscopy showed stenosis, edema, congestion in the distal airway of tracheostomy tube (white arrow).

After hospital admission, the patient was immediately provided with invasive ventilator-assisted ventilation. However, his difficulty in breathing was not relieved. The blood oxygen saturation remained between 60% and 80%. The ventilator alarm indicated high airway resistance and the nurses were unable to insert the suction pipes into the airway. Immediate fiberoptic bronchoscopy showed diffuse edema and stenosis of the inferior tracheal airways (Fig. 2).

Given that stent implementation and other treatment strategies were not supplied in this critical situation, we first selected a particular cannula size specific for obese patients. However, the cannula was not long enough to completely expand the narrowed airway. For this reason, tracheal intubation was used to temporarily replace the tracheotomy cannula, which successfully expanded the narrowed airways. The blood oxygen saturation returned to normal, and the symptoms were quickly relieved. After 2 days of treatment and observation in our department, the patient was stable and transferred to the pulmonary and critical care department for further treatment of airway stenosis. Supporting ventilation was withdrawn prior to the transfer.

## 4 Discussion

Central airway stenosis is an urgent and critical illness not commonly seen in clinical practice. However, it is essential that physicians are unaware of this condition, as it can be life-threatening if not diagnosed correctly in a timely manner. The pathogenesis of central airway stenosis is primarily related to bacterial infections, scar tissue, granulation tissue formation, foreign bodies, tumors, and specific types of trauma. In this case, the bronchoscope showed diffuse airway edema. Formation of the edema was associated with physical stimulation, repeated sputum aspiration, and a chronic bacterial infection due to the long-term opening of the airways. The presence of bacterial biofilms, increased bacterial counts, and overexpression of transforming growth factor-β(TGF-beta) and SMAD3 may also be associated with airway stenosis.^[1,2]^

There are several options for treating central airway stenosis, including rigid bronchoscopy, bronchoscopic high-power laser therapy, high-frequency electric needle knife, and balloon-expanding stents. Microwave coagulation therapy, argon plasma coagulation, and laser thermal coagulation techniques are optimal for treating patients with airway stenosis caused by tumors. When applied under a high oxygen concentration, the cryosurgical unit ERBE-CRYO2 can reduce the risk of airway fires.^[3]^ Airway stents are also useful for treating airway stenosis and fistulas. In a previous study, Oki and Saka demonstrated that airway stenting could improve the overall survival of patients with small cell lung cancer (SCLC).^[4]^ Stenting implementation facilitates extubation and reduces the risk of obstruction in critically ill patients with malignant central airway stenosis.^[5,6]^ In medical emergencies, interventional bronchoscopy for airway obstructions could relieve the symptoms, facilitate palliative chemotherapy, and potentially increase the overall survival of patients.^[7]^ There are other effective strategies for treating malignant airway stenosis, such as staging radioactive particle implantation guided by CT and fiber bronchoscopy.^[8]^ Lastly, photodynamic therapy (PDT) with chemotherapy was found to be a safe and effective treatment for patients with bronchial obstructions.^[9]^

As mentioned above, interventional bronchoscopy can relieve the symptoms and improve the survival of patients with emergent airway obstructions.^[4]^ However, interventional bronchoscopy is not available in some hospitals. It is also difficult to implement quickly in emergent situations occurring at night. In this case, a common tracheal intubation catheter was used to replace the tracheotomy cannula, which had a satisfactory rescue effect for our patient with severe central airway stenosis after tracheotomy. This procedure aided the patient in surviving the most dangerous phase of the condition and allowed him to the opportunity to receive proper and timely treatment for this life-threatening condition.

Some problems may occur after replacing tracheal intubation cannulas with conventional endotracheal cannulas. First, it may be difficult to fix the tracheal intubation cannula when it is placed in the position of the tracheotomy cannula. Tracheal stenosis may recur if fixation is unstable. Second, the tracheal intubation was longer than the tracheal cannula, which made it more difficult for the patient to spontaneously expel sputum than before. However, these problems can be solved with proper actions, such as strengthening fixation and sputum aspiration.

## 5 Conclusion

In conclusion, this case has positively impacted the treatment of patients with similar conditions. The conventional endotracheal cannula is useful for patients with central airway stenosis, in addition to patients with a tracheotomy cannula as it relieves the obstructive symptoms and increases overall patient survival.

## Author contributions

Hai-feng Li designed/performed most of the investigation, data analysis and wrote the manuscript; Bao-peng Xing and Li-sha Song provided pathological assistance; Wei Wang and Bao-hua Liu contributed to interpretation of the data and analyses. All of the authors have read and approved the manuscript.

**Conceptualization:** Hai-Feng Li, Bao-Peng Xing, Li-Sha Song, Wei Wang.

**Data curation:** Hai-Feng Li, Bao-Peng Xing, Li-Sha Song.

**Formal analysis:** Hai-Feng Li, Li-Sha Song.

**Funding acquisition:** Li-Sha Song, Wei Wang.

**Investigation:** Wei Wang.

**Methodology:** Li-Sha Song, Wei Wang, Bao-Hua Liu.

**Project administration:** Bao-Peng Xing, Li-Sha Song, Wei Wang.

**Resources:** Hai-Feng Li, Bao-Peng Xing, Bao-Hua Liu.

**Software:** Hai-Feng Li, Bao-Peng Xing, Wei Wang, Bao-Hua Liu.

**Supervision:** Bao-Peng Xing, Bao-Hua Liu.

**Validation:** Bao-Hua Liu.

**Writing – review & editing:** Wei Wang.
